# Whole cell biosynthesis of a functional oligosaccharide, 2′-fucosyllactose, using engineered *Escherichia coli*

**DOI:** 10.1186/1475-2859-11-48

**Published:** 2012-04-30

**Authors:** Won-Heong Lee, Panchalee Pathanibul, Josh Quarterman, Jung-Hyun Jo, Nam Soo Han, Michael J Miller, Yong-Su Jin, Jin-Ho Seo

**Affiliations:** 1 Department of Food Science and Human Nutrition, University of Illinois at Urbana-Champaign, Urbana, IL, 61801, USA; 2 Institute for Genomic Biology, University of Illinois at Urbana-Champaign, Urbana, IL, 61801, USA; 3 Department of Food Science and Technology, Chungbuk National University, Cheongju, 361-763, Korea; 4 Department of Agricultural Biotechnology and Center for Agricultural Biomaterials, Seoul National University, Seoul, 151-921, Korea

**Keywords:** Recombinant *Escherichia coli*, GDP-l-fucose, α-1,2-fucosyltransferase, 2′-fucosyllactose, Elementary flux mode analysis

## Abstract

**Background:**

2'-Fucosyllactose (2-FL) is a functional oligosaccharide present in human milk which protects against the infection of enteric pathogens. Because 2-FL can be synthesized through the enzymatic fucosylation of lactose with guanosine 5′-diphosphate (GDP)-l-fucose by α-1,2-fucosyltransferase (FucT2), an 2-FL producing *Escherichia coli* can be constructed through overexpressing genes coding for endogenous GDP- l-fucose biosynthetic enzymes and heterologous fucosyltransferase.

**Results:**

The gene for FucT2 from *Helicobacter pylori* was introduced to the GDP- l-fucose producing recombinant *E. coli* BL21 star(DE3) strain. However, only small amount of 2-FL was produced in a batch fermentation because the *E. coli* BL21star(DE3) strain assimilated lactose instead of converting to 2-FL. As an alternative host, the *E. coli* JM109(DE3) strain which is incapable of assimilating lactose was chosen as a 2-FL producer. Whole cell biosynthesis of 2-FL from lactose was investigated in a series of batch fermentations using various concentrations of lactose. The results of batch fermentations showed that lactose was slowly assimilated by the engineered *E*. *coli* JM109(DE3) strain and 2-FL was synthesized without supplementation of another auxiliary sugar for cell growth. A maximum 2-FL concentration of 1.23 g/l was obtained from a batch fermentation with 14.5 g/l lactose. The experimentally obtained yield (g 2-FL/g lactose) corresponded to 20% of the theoretical maximum yield estimated by the elementary flux mode (EFM) analysis.

**Conclusions:**

The experimental 2-FL yield in this study corresponded to about 20% of the theoretical maximum yield, which suggests further modifications via metabolic engineering of a host strain or optimization of fermentation processes might be carried out for improving 2-FL yield. Improvement of microbial production of 2-FL from lactose by engineered *E. coli* would increase the feasibility of utilizing 2-FL as a prebiotic in various foods.

## Background

Human milk oligosaccharides (HMOs) are known to be the most relevant factor for the development of intestinal microbiota in breast-fed infants [1]. Also, HMOs have been reported to play important roles in preventing adhesion of pathogens and toxins to epithelial surfaces [2]. Fucosyloligosaccharides, such as 2′-fucosyllactose, lacto-*N*-fucopentaose and lacto- *N*-difucohexaose, are common HMOs. Fucosylated oligosaccharides act as growth stimulating factors for select Bifidobacteria and soluble analogs of receptors for pathogenic bacteria, thereby protecting infants against infection from enteric pathogens and binding of toxins [3,4]. Specifically, α-1,2-linked fucosylated oligosaccharides are reported to exhibit protective activity against several pathogens including *Campylobacter jejuni*[3,5], *Salmonella* enteric serotype *Typhimurium*[6], Enterotoxigenic *E. coli*[7], *Helicobacter pylori*[8] and noroviruses [9]. Among them, 2′-fucosyllactose (2-FL) is the most abundant fucosyloligosaccharide in human milk and accounts for more than 30% of total HMOs [3,5]. Low levels of 2-FL in the milk of sore mothers have been reported to be associated with a higher rate of diarrhea in breast-fed infants [3]. Hence, 2-FL is a promising oligosaccharide for nutraceutical and pharmaceutical purposes.

**Figure 1:**
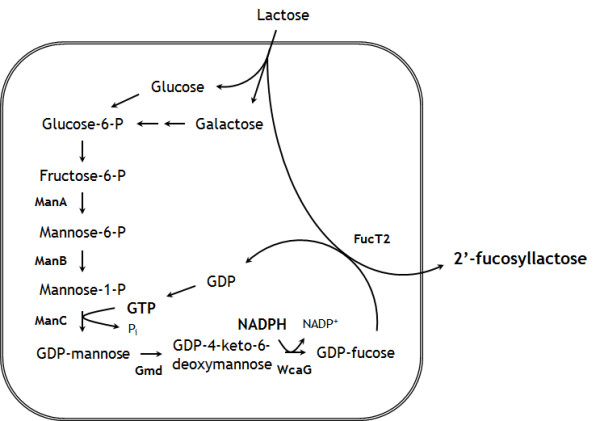
**The metabolic pathway for GDP--fucose and 2′-fucosyllactose (2-FL) biosynthesis in recombinant*E. coli*.** The names of enzymes are abbreviated as follows; ManA, mannose 6-phosphate isomerase; ManB, phosphomannomutase; ManC, mannose 1-phosphate guanylyltransferase; Gmd, GDP- d-mannose-4,6-dehydratase; WcaG, GDP-4-keto-6-deoxymannose 3,5-epimerase 4-reductase; FucT2, α-1,2-fucosyltransferase. Pi, GDP and GTP denote phosphate, guanosine 5′-diphosphate and guanosine 5′-triphosphate.

2-FL can be synthesized through the enzymatic fucosylation of lactose by α-1,2 fucosyltransferase (FucT2), which requires guanosine 5′-diphosphate (GDP)-l-fucose as a donor of l-fucose [10]. * Escherichia coli* is known to be able to synthesize GDP- l-fucose since GDP- l-fucose is used for biosynthesis of colanic acid, one of the main components of the cell wall [11]. Therefore, 2-FL can be produced via engineering of the GDP-l-fucose biosynthetic pathway and overexpression of the fucosyltransferase gene in metabolically engineered *E*. *coli*. Figure 1 shows the metabolic pathway for biosysnthesis of GDP-l-fucose and 2-FL in recombinant *E*. *coli*.

Previously, biosynthesis of fucosyloligosaccharides using a recombinant microorganism and fucosyltransferase has been reported. The enzymatic synthesis of 2-FL was examined by using purified FucT2, GDP-l-fucose and lactose [10], however, the high cost of GDP-l-fucose and FucT2 purification may be a limiting factor for large-scale production of fucosyloligosaccharides. Production of several fucose-containing lacto-oligosaccharides in recombinant *E. coli* was also reported through simultaneous overexpression of fucosyltransferase and the regulatory protein for colanic acid biosynthesis [12,13], which suggested that whole cell synthesis of fucosyloligosaccharides through direct amplification of the GDP-l-fucose biosynthesis might be feasible.

To construct an efficient 2-FL production system by metabolic engineering, an understanding and detailed analysis of a cellular metabolic network involved in the 2-FL biosynthesis is important. Elementary flux mode (EFM) analysis has emerged as a powerful tool for metabolic pathway analysis. EFM analysis is a useful mathematical tool for defining and describing all metabolic routes that are both stoichiometrically and thermodynamically feasible for a group of enzymes. The EFM analysis can decompose a complex metabolic network of many highly interconnected reactions into uniquely organized pathways that support steady state of metabolism. EFM analysis can provide identification of all genetically independent pathways, determination of the most efficient physiological state of a cell, and analysis of metabolic network properties such as robustness and regulation [14-16]. Hence, it can be a useful tool for understanding dynamics of cellular metabolism and rational design of the host strain’s metabolism for 2-FL production.

We have previously developed a recombinant *E*. *coli* system for efficient production of GDP- l-fucose by metabolic engineering. An enhancement of GDP- l-fucose production was achieved by modulation of several factors for biosynthesis of GDP- l-fucose such as amplification of GDP- d-mannose biosynthesis, regeneration of NADPH and manipulation of the guanosine nucleotides biosynthetic pathway [17-19].

In the present study, the GDP-l-fucose production system was applied for efficient production of 2-FL by introduction of the FucT2 gene from *Helicobacter pylori* into the recombinant *E. coli* able to overproduce GDP- l-fucose. Whole cell biosynthesis of 2-FL from lactose was assessed in a series of batch fermentations for recombinant *E. coli* overexpressing the necessary genes for GDP- l-fucose production and the FucT2. An EFM analysis for 2-FL production in the recombinant *E. coli* was used to compare and evaluate experimental results.

## Methods

### Strains and plasmids

*E. coli* TOP10 [F- *mcr*A Δ( *mrrhsd*RMS- *mcr*BC) φ80 *lac*ZΔM15 Δ *lac*X74 *rec*A1 *ara*D139 Δ( *araleu*) 7697 *gal*U *gal*K *rps*L (Str^R^) *end*A1 *nup*G] was used for genetic manipulation. *E*. *coli* BL21star(DE3) [F^−^*omp*T, *hsd*SB(r_B_^−^m_B_^−^), *galdcm rne131* (DE3)] (Invitrogen, Carlsbad, CA, USA) and JM109(DE3) *end*A1 *gln*V44 *thi*-1 *rel*A1 *gyr*A96 *rec*A1 *mcr*B^+^ Δ(*lac-pro*AB) e14- [F' *tra*D36 *pro*AB^+^*lacI*^q^*lac*ZΔM15] *hsd*R17(r_K_^-^m_K_^+^) (DE3)] (NEB, Ipswich, MA, USA) were used for production of GDP-l-fucose and 2-FL. Plasmid pmBCGW containing the polycistronic *gmdwcaG* gene cluster and *manBmanC* gene cluster was previously constructed using plasmid pETDuet-1 [18]. The gene encoding FucT2 was obtained by the polymerase chain reactions (PCR) using the genomic DNA of the *Helicobacter pylori* 26695 strain (ATCC 700392) as template [20]. Two PCR primers of fucT2_F and fucT2_R were used for the amplification of the *fucT2* gene. After digestion of PCR fragments of the *fucT2* gene and pCOLADuet-1 (Merck Biosciences, Darmstadt, Germany) with *Nco*I and *Sac*I, the DNA fragments were ligated to construct plasmid pHfucT2. Plasmids and primers used in this work are listed in Table 1. The constructed plasmid was confirmed by DNA sequencing. The conditions for PCR reaction, DNA manipulation and bacterial transformation followed the descriptions in the previous study [21].

**Table 1:** List of primers and plasmids used in this study

**Name**	**Sequence of PCR primers and description for plasmids**	**Source**
PCR primers		
fucT2_F (*Nco*I)	5′-ACATGCCATGGCTTTTAAGGTGGTGCAA-3′	*H*. *pylori* 26695 (ATCC 700392)
fucT2_R (*Sac*I)	5′-AGTCCGAGCTCTTAAGCGTTATACTTTTGGGA-3′	
Plasmids		
pETDuet-1	two T7 promoters with two MCS, pBR322 replicon (copy number ~40), Amp^r^	Merck Biosciences
pCOLADuet-1	two T7 promoters with two MCS, ColA replicon (copy number 10 ~ 12), Kan^r^	Merck Biosciences
pmBCGW	derived from pETDuet-1, P_T7_-*manB-manC* ( *Nco*I/ *Sac*I)-P_T7_-*gmd-wcaG* ( *Nde*I/ *Xho*I)-T_T7_, Amp^r^	Lee et al., 2009
pHfucT2	derived from pCOLADuet-1, P_T7_- *fucT2* ( *Nco*I/ *Sac*I)-P_T7_-MCS2-T_T7_, Kan^r^	this study

### Batch fermentation

Batch fermentation was carried out in a 250 ml flask containing 50 ml of LB medium at 25°C and pH 6.8. Agitation speed was maintained at 250 rpm. When dry cell mass reached 0.3 g/l, 0.1 mM isopropyl-β-d-thiogalactopyranoside (IPTG) was added to culture broth. After 3 h of additional cultivation, 2.6 g/l (or 14.5 g/l) lactose was added for 2-FL production.

### Analytical methods

Cell concentration was measured by optical density (OD) at 600 nm using a spectrophotometer (Biomate 5, Thermo, NY, USA). Overexpression of FucT2 inside the cell was analyzed by using sodium dodecyl sulfate-polyacrylamide gel electrophoresis (SDS-PAGE, 12% polyacrylamide). After 3 h of 0.1 mM IPTG induction, cells were collected and the concentration was adjusted to around 7.2 g/l. They were resuspended in 50 mM potassium phosphate buffer (pH 7.0) and disrupted by an ultrasonic processor. After centrifugation at 15,000 × g for 20 min, the supernatant (soluble fraction) and debris (insoluble fraction) were separated. Ten microliters of the soluble protein fraction (approximately 0.04 mg) and the same volume of the total and insoluble protein fractions were subjected to SDS-PAGE. Gels were stained with Coomassie brilliant blue solution and images were analyzed using a densitometer.

Concentrations of lactose, 2-FL and acetate in batch fermentations were determined by using a high performance liquid chromatography (HPLC) system (Agilent Technologies 1200 Series) equipped with a Rezex ROA Organic Acid H^+^ column (Phenomenex, Torrance, CA, USA) and a refractive index (RI) detector (Agilent, Palo Alto, CA, USA). The column was eluted with 0.01 N H_2_SO_4_ at a flow rate of 0.6 ml/min at 50°C.

In order to confirm 2-FL biosynthesis, culture broth at the end of the batch fermentation was collected and analyzed using a liquid chromatography/mass spectrometry (LC/MS) system. The LC (Agilent Technologies 1100 Series) was equipped with an Agilent Zorbax Eclipse ZDB-C8 (4.6x150 mm, 5 μm) column and an Agilent LC/MSD Trap XCT Plus detector. The column was eluted at a flow rate of 0.4 ml/min by the following gradient program: 95% (v/v) eluent A (15 mM ammonium acetate) and 5% eluent B (acetonitrile) for 1 min; 5% to 95% eluent B over 6 min; 95% eluent B over 10 min. The scan range for MS was 70–600 mass-to-charge ratio (m/z).

### Construction of metabolic network model for *E. coli* producing 2-FL from lactose

A metabolic network model was constructed for 2-FL producing *E*. *coli* that grows on lactose. The *E. coli* network was based on a model that was introduced by Stelling et al. [22] to examine the relationship between structure and function in metabolic networks. Furthermore, the model has been used for calculating elementary flux modes in previous reports [23,24]. The metabolic network was composed of 108 reactions, which were involved in carbon central metabolism, amino acid synthesis, fatty acid synthesis and biomass production (Additional file 1). The catabolic part of the model included substrate uptake reactions, glycolysis, pentose phosphate pathway, TCA cycle, and excretion of by-products (e.g. acetate, formate, lactate, and ethanol). Previous networks were extended to include the anaplerotic reactions (e.g. malic enzyme and pyruvate oxidase) in addition to parallel pathways for initial acetate metabolism. The anabolic part of the model covers the conversion of precursors into building blocks like macromolecules and biomass. The core *E. coli* model from Stelling et al. [22] was modified in this research to account for lactose consumption and synthesis of 2-FL. Among the reactions added for 2-FL synthesis, some minor adjustments were made to simplify the model. Lactose was assumed to break down to 2 moles of glucose because galactose can be easily converted into glucose-6-phosphate. ATP was used in place of GTP for energy transfer. As for the mass balance, it should be noted that ADP is formed whenever ATP is consumed for all the metabolic reactions in the network. The mass balance equation on ATP is therefore the negative of the mass balance on ADP and thus the two equations are linearly dependent. Therefore, ADP can be excluded from the model in order to simplify the subsequent EFM calculation. The same is true for other cofactor pairs like NADP/NADPH and NAD/NADH. The EFM pathways in the model were estimated using METATOOL 5.1 [14,16] with Matlab.

## Results

### Expression of α-1,2-fucosyltransferase (FucT2) in recombinant *E. coli*

**Figure 2:**
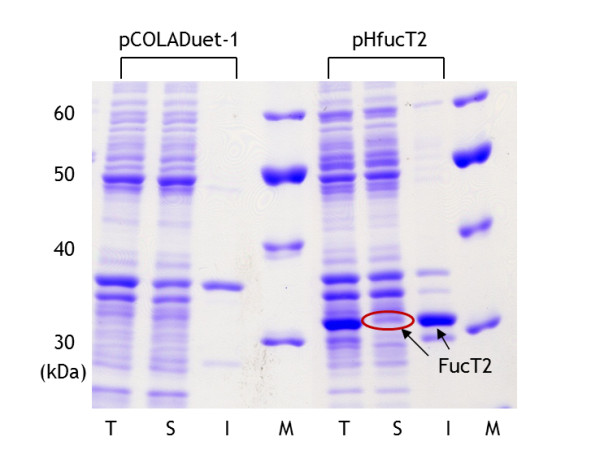
**SDS-PAGE analysis of the cell crude extract of recombinant*E. coli*BL21star(DE3) strains harboring pCOLADuet-1 and pHfucT2, respectively.** Cells were harvested after 3 h of 0.1 mM IPTG induction. T, S and I denote total, soluble and insoluble protein fractions, respectively. The arrow indicates the corresponding protein band with the estimated molecular weight of FucT2. Lane M indicates size marker.

The expression pattern of FucT2 was investigated during a batch fermentation of recombinant *E*. *coli* harboring plasmid pHfucT2. In order to maximize the expression of the soluble form of FucT2 in the recombinant *E. coli*, 0.1 mM of IPTG was used. As shown in Figure 2, a 33 kDa protein (consistent with FucT2, [20]) was found in both soluble and insoluble fraction. While a significant amount of FucT2 was expressed in inclusion bodies, biosynthesis of 2-FL was expected because a soluble form of FucT2 was available as well.

### Batch fermentations

From the preliminary experiments (whole cell bioconversion of 2 g/l lactose with *E*. *coli* BL21star(DE3) strain), we concluded that the *E*. *coli* BL21star(DE3) strain is not beneficial for 2-FL production because it consumed lactose for growth and maintenance instead of converting to 2-FL (data not shown). Some *E*. *coli* strains, such as DH5α and JM series, are known to be unable to assimilate lactose or utilize lactose extremely inefficiently due to partial deletion of the *lac*Z gene, which codes for β-galactosidase. As such, these *E. coli* strains might be useful for 2-FL production. Hence, *E*. *coli* JM109(DE3) enabling overexpressing proteins under the control of *T7* promoter was chosen as an alternative host stain for 2-FL production.

**Figure 3:**
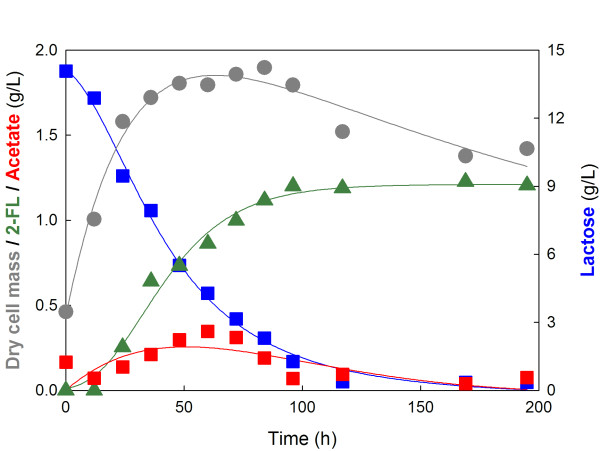
**Profile of 2-FL production in the batch fermentation of recombinant*E. coli*JM109(DE3) strain harboring plasmids pmBCGW and pHfucT2.** After 3 h of 0.1 mM IPTG induction, 14.5 g/l of lactose was added for 2-FL production. Symbols denote as follows; *grey circle*, dry cell mass; *green triangle*, 2-FL concentration; *blue square*, lactose concentration; *red square*, acetate concentration. Measurement of cell, lactose, acetate and 2-FL concentrations were done by three independent experiments. Symbols in the figure show the representative values of the batch fermentations.

2-FL production for BL21star(DE3) and JM109(DE3) was compared under batch fermentation conditions. In order to allow sufficient production of both GDP-l-fucose biosynthetic enzymes and FucT2 inside the cells, the cells were cultivated for 3 h after 0.1 mM IPTG induction. Then, 2.6 g/l of lactose was added to initiate 2-FL production without addition of additional sugar because GDP- l-fucose can be produced from LB media [17,18]. During the fermentations, extracellular 2-FL production (in the medium) was monitored by HPLC analysis. As a result, a small amount of 2-FL (10 mg/l) was produced in the batch fermentation of recombinant *E*. *coli* BL21star(DE3). Meanwhile, much higher amount of 2-FL was produced in the batch fermentation of recombinant *E. coli* JM109(DE3). About 140 mg/l of 2-FL was produced from 2.6 g/l of lactose while 0.4 g/l of lactose remained unused at the end of the fermentation (data not shown). These results suggest that the lactose concentration should be controlled at more than 0.5 g/l to maintain 2-FL production. Consequently, a yield of 60 mg 2-FL/g lactose was obtained from the batch fermentation of *E*. *coli* JM109(DE3) when 2.6 g/l of lactose was used. In order to obtain a higher amount of 2-FL, a batch fermentation with a higher concentration of lactose was carried out. Figure 3 shows the profiles of lactose consumption and 2-FL production in the batch fermentation of recombinant *E*. *coli* JM109(DE3) with 14.5 g/l lactose. The cells consumed lactose slowly but produced 2-FL constantly for 96 h. After 96 h of fermentation, the 2-FL concentration did not increase any further and lactose consumption stopped. As a result, a maximum 2-FL concentration of 1.23 g/l was obtained, which corresponded to a nine-fold (1.23 g/l vs. 140 mg/l) increase as compared with the previous fermentation with 2.6 g/l lactose. 2- FL yield increased to 90 mg 2-FL/g lactose when 14.5 g/l of lactose was used. This improvement might be caused from increased lactose availability inside the cell. The results of the batch fermentations are summarized in Table 2.

**Table 2:** **Summary of batch fermentations of*E*.*coli*strains producing 2-FL from lactose**

**Strains**	**Plasmids**	**Initial lactose concentration (g/l)**	**Maximum dry cell mass (g/l)**	**Maximum 2-FL concentration (g/l)**	**Yield (g 2-FL/g lactose)**
BL21star(DE3)	pmBCGW + pHfucT2	2.56 ± 0.04	1.84 ± 0.05	0.01 ± 0.001	0.005 ± 0.001
JM109(DE3)	pmBCGW + pHfucT2	2.55 ± 0.02	1.17 ± 0.05	0.14 ± 0.015	0.06 ± 0.005
		14.54 ± 0.67	1.70 ± 0.28	1.23 ± 0.011	0.09 ± 0.004

### Confirmation of 2-FL biosynthesis by recombinant *E. coli* overexpressing GDP- l-fucose biosynthetic enzymes and FucT2

**Figure 4:**
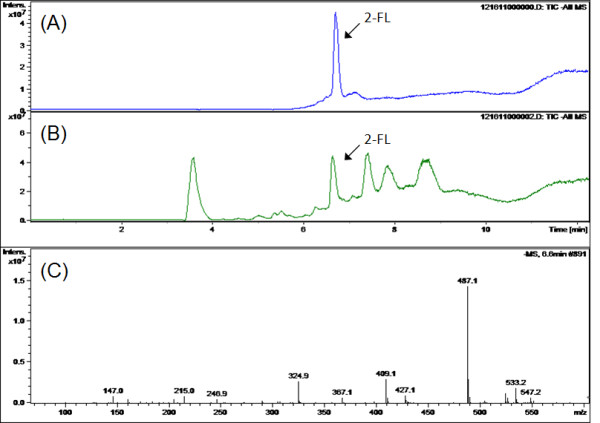
**LC/MS analysis of 2-FL biosynthesis in the batch fermentation of the recombinant*E*.*coli*JM109(DE3) overexpressing ManB, ManC, Gmd, WcaG and FucT2.** At the end of batch fermentation, culture broth was collected for confirmation of extracellular 2-FL production. HPLC analysis of 100 mg/l 2-FL standard solution ( **A**), HPLC analysis of culture broth of *E*. *coli* JM109(DE3) harboring pmBCGW + pHfucT2 ( **B**) and MS analysis of the compound with the retention time = 6.6 min in the culture broth of *E*. *coli* JM109(DE3) harboring pmBCGW + pHfucT2 ( **C**).

LC/MS analysis was performed to confirm the biosynthesis of 2-FL in the recombinant *E*. *coli* JM109(DE3) strain overexpressing ManB, ManC, Gmd, WcaG and FucT2. HPLC data showed that a compound with the identical retention time to 2-FL was detected in the culture broth (Figure 4B). MS scanning data (compound with RT = 6.6 min) showed ion fragment of m/z 487.1, which is compatible with 2-FL (Figure 4C).

### Evaluation of 2-FL yield using EFM analysis for 2-FL producing *E. coli* from lactose

**Figure 5:**
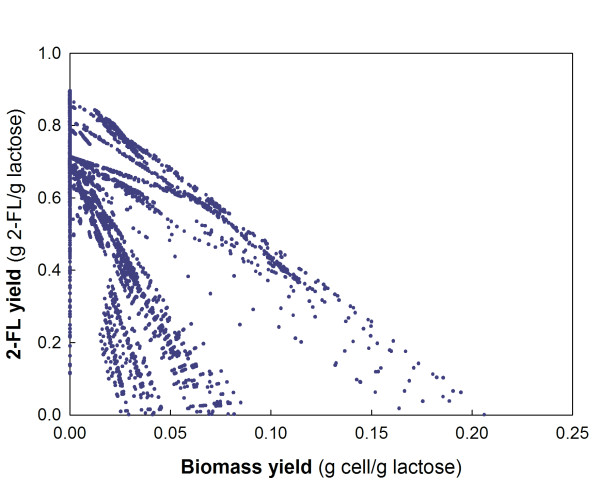
**Calculation of the theoretical maximum yield of 2-FL from lactose. ** Elementary flux mode (EFM) analysis was carried out for 2-FL producing *E*. *coli.*

In order to evaluate the efficiency of 2-FL production from lactose using the recombinant *E*. *coli* JM109(DE3) strain, elementary flux mode (EFM) analysis was employed to estimate a maximum theoretical yield of 2-FL from lactose. Figure 5 shows the prediction of theoretical 2-FL yield versus biomass yield for *E*. *coli* growing on lactose. Our experimental result from a batch fermentation of 14.5 g/l of lactose resulted in a biomass yield of 0.1 g biomass/g lactose. This suggests that 2-FL production from lactose by the engineered *E. coli* reached 20% of the maximum 2-FL production capacity.

## Discussion

In human milk, α-1,2-fucosylated oligosaccharides such as 2-FL, are known to have protective activity against pathogenic bacteria and their toxins [3,5,9]. Hence, the availability of large amounts of α-1,2-fucosylated oligosaccharides would be useful in ready-to-use materials, drugs for fundamental investigations or therapeutic purposes. As biosynthesis of GDP-l-fucose, a key compound for biosynthesis of α-1,2-fucosylated oligosaccharides, requires a number of enzymes and cofactors such as NADPH and GTP, a whole-cell conversion approach might be more realistic for industrial production than other chemical or enzymatic approaches [25].

In previous studies, improvements of GDP-l-fucose production in recombinant *E. coli* were attempted by manipulating the pathways enabling GDP- l-fucose biosynthesis from glucose [17-19]. This study was undertaken to upgrade the GDP-l-fucose production system for 2-FL production through additional overexpression of FucT2. To this end, the *fucT2* gene from *H*. *pylori* was cloned and overexpressed in the *E*. *coli* BL21star(DE3) strain. While an insoluble form of FucT2 was a major expression form, an active FucT2 was also observed in the recombinant *E*. *coli* as shown in Figure 2. With the aid of FucT2 overexpression, recombinant *E*. *coli* BL21star(DE3) could produce 2-FL in the batch fermentation with 2.6 g/l lactose. However, 2-FL yield was fairly low (5 mg 2-FL/g lactose). *E*. *coli* BL21star(DE3) seemed to assimilate lactose instead of converting to 2-FL. Most of the initially added lactose was consumed within 12 h of fermentation with a marginal growth during the fermentation (data not shown). These results suggested that *E*. *coli* BL21star(DE3) is not appropriate for 2-FL production.

Previously, several attempts for production of fucosylated (or sialylated) oligosaccharides from lactose have been made using the derivative of *E*. *coli* JM107 and JM109 since these strains are unable to produce an active β-galactosidase due to the insertion of the M15 single strand DNA into the *lacZ* gene [12,13,26,27]. In these cases, glucose (or glycerol) was used as another carbon source for GDP-fucose production (or CMP-*N*-acetylneuraminic acid). These nucleotide sugars are subsequently used for fucosylation (or sialylation). According to the previous reports, *E*. *coli* JM109(DE3) was chosen as an alternative host strain for 2-FL production. As expected, the use of *E*. *coli* JM109(DE3) allowed production of a considerable amount of 2-FL in the batch fermentation, corresponding to a 14-fold increase in 2FL concentration compared with the value obtained in BL21star(DE3) strain. However, a theoretical yield of 2-FL from lactose was predicted to be 1.4 g 2-FL/g lactose when cells cannot utilize lactose for growth. Meanwhile, our experimental yield of 2-FL was only about 0.1 g 2-FL/g lactose. This result indicates that more than 90% of lactose consumed was used for other purposes such as biomass production and endogeneous metabolism. Slow consumption of lactose was also observed in the batch fermentation with mixed sugars (2 g/l of lactose and 5 g/l of mannose), where we expected that lactose could be mainly used for 2-FL production as mannose might be used for cell growth. Although an enhancement of 2-FL yield (0.13 g 2-FL/g lactose) was obtained, most of the consumed lactose was not used for 2-FL production (data not shown). It is probable that incompletely inactivated β-galactosidase or cryptic β-galactosidase inside the cell might cause the reduction of 2-FL yield from lactose. Insertion of λ(DE3) into the genome might cause the slow assimilation of lactose, which was supported by a previous report that α-complementation of LacZ was observed with the insertion of the DE3 cassette into the genomic DNA of *E*. *coli* JM101 [26].

Since slow consumption of lactose and production of biomass were observed in the batch fermentation with 2.6 g/l lactose, another batch fermentation with a high concentration of lactose (14.5 g/l) was conducted in order to obtain a higher amount of 2-FL. Generally, *E*. *coli* is known to produce acetate when growing on an excessive sugar even under aerobic conditions. However, JM109(DE3) strain showed no acetate production even when excessive amounts (14.5 g/l) of lactose were added as displayed in Figure 3. It is generally known that acetate formation is accelerated when the metabolic fluxes to pyruvate exceed the capacity of the respiratory metabolism [28,29]. Slow consumption of lactose might not lead to acetate formation, suggesting that the lactose utilization rate by *E. coli* JM109(DE3) is not fast enough to cause acetate formation. This result could be beneficial for designing a 2-FL production process such as a fed-batch type operation since acetate formation is known to be one of the main problems occurring in fed-batch type operations of *E*. *coli*[17]. However, low yield and productivity of 2-FL from lactose will need to be improved for industrial applications. The 2-FL production system in this study was verified by comparing the experimental 2-FL yield with the theoretical maximum yield estimated by the EFM analysis. 2-FL yield corresponded to about 20% of the theoretical maximum yield, which suggests further modifications via metabolic engineering of a host strain or optimization of fermentation processes should be carried out for improvement of 2-FL yield. Increased FucT2 solubility and intracellular lactose availability may be considered as primary approaches for improvement of 2-FL yield.

## Conclusions

In this study, construction of efficient 2-FL production system was attempted. The *fucT2* gene from *H. pylori* was introduced into the recombinant *E. coli* able to overproduce GDP- l-fucose and biosynthesis of 2-FL was observed in the batch fermentation with lactose. The 2-FL production system using the *E*. *coli* JM109(DE3) strain showed a low rate of lactose assimilation and produced a considerable amount of 2-FL in the simple batch fermentation without acetate formation. The experimental 2-FL yield corresponded to 20% of the theoretical maximum yield, which indicates that more research should be conducted. Efficient microbial 2-FL production may be useful for utilizing 2-FL as a nutraceutical compound for various applications.

## **Competing interests**

The authors declare that they have no competing interests.

## **Authors’ contributions**

WHL, YSJ and JHS designed research. WHL, PP, JQ and JHJ performed the experiments. NSH and MJM contributed general advice and materials. WHL, PP, JQ, YSJ and JHS analyzed data and wrote the manuscript. YSJ and JHS supervised all works. All authors read and approved the final manuscript.

## Supplementary Material

Additional file 1 METATOOL input file. Click here for file
