# Supplementary material for: Whole cell biosynthesis of a functional oligosaccharide, 2′-fucosyllactose, using engineered Escherichia coli
Source: Microb Cell Fact. 2012 Apr 30;11:48. doi: 10.1186/1475-2859-11-48 (PMC3442965; doi:10.1186/1475-2859-11-48)
Supplement: Additional file 1 — METATOOL input file. [file 1475-2859-11-48-S1.doc]

-ENZREV

CO2_ex G6P::F6P F16P::T3P DHAP::G3P G3P::DPG DPG::3PG 3PG::2PG 2PG::PEP Cit::ICit

ICit::alKG SuccCoA::Succ Fum::Mal Mal::OxA G6P::PGlac AcCoA::Adh Adh::Eth Rl5P::X5P

Rl5P::R5P Transket1 Transaldo Transket2 AcCoA::AcP AcP::Ac Pyr::Lac NADHDehydro

TransHydro ATPSynth MTHF_Synth

-ENZIRREV

mue O2_up N_up S_up Glc_PTS Glc_ATP DHAP::Glyc3P Lac_ex Lact_up Lactase FL_rxn Eth_ex Ac_ex Form_ex F16P::F6P F6P::F16P PEP::PYR Pyr::PEP PYR::AcCoA AcCoA::Cit alKG::SuccCoA Succ::Fum Fum::Succ ICit::Glyox Glyox::Mal PGlac::PGluc PGluc::Rl5P OxA::PEP PEP::OxA Pyr::Form Oxidase ATPdrain Chor_Synth PRPP_Synth Ala_Synth Val_Synth Leu_Synth Asn_Synth_1 Asp_synth Asp::Fum Asp::AspSAld AspSAld::HSer Lys_Synth Met_Synth Thr_Synth Ile_Synth His_Synth Glu_synth Gln_Synth Pro_Synth Arg_Synth Trp_Synth Tyr_Synth Phe_Synth Ser_Synth Gly_Synth Cys_Synth rATP_Synth rGTP_Synth rCTP_Synth rUTP_Synth dATP_Synth dGTP_Synth dCTP_Synth dTTP_Synth mit_FS_Synth UDPGlc_Synth CDPEth_Synth OH_myr_ac_Synth C14_0_FS_Synth CMP_KDO_Synth NDPHep_Synth TDPGlcs_Synth UDP_NAG_Synth UDP_NAM_Synth di_am_pim_Synth ADPGlc_Synth Mal::Pyr Pyr::Ac Ac::AcCoA

-METINT

Gluc Lact G6P F6P F16P DHAP Glyc3P G3P DPG 3PG 2PG PEP Pyr AcCoA Cit ICit alKG SuccCoA Succ Fum Mal OxA Glyox R5P Rl5P E4P X5P S7P PGlac PGluc ATP NADH NADPH QH2 Hp O2 CO2 N S AcP Ac Form Lac Adh Eth Chor PRPP MTHF AspSAld HSer Ala Cys Asp Glu Phe Gly His Ile Lys Leu Met

Asn Pro Gln Arg Ser Thr Val Trp Tyr rATP rGTP rCTP rUTP dATP dGTP dCTP dTTP mit_FS UDPGlc

CDPEth OH_myr_ac C14_0_FS CMP_KDO NDPHep TDPGlcs UDP_NAG UDP_NAM di_am_pim ADPGlc

-METEXT

O2_ext N_ext CO2_ext Lac_ext Lact_ext Eth_ext Ac_ext Form_ext ATP_ext FL_ext Biomass

-CAT

mue : 0.14176 Glyc3P + 26.2949 ATP + 0.60097 Ala + 0.10124 Cys + 0.26647 Asp + 0.30747 Glu + 0.2048 Phe + 0.67725 Gly + 0.10473 His + 0.32116 Ile + 0.37935 Lys + 0.49804 Leu + 0.16989 Met + 0.26647 Asn + 0.24436 Pro + 0.29091 Gln + 0.32698 Arg + 0.38031 Ser + 0.28044 Thr + 0.46778 Val + 0.062835 Trp + 0.15244 Tyr + 0.1489 rATP + 0.18319 rGTP + 0.11366 rCTP + 0.12273 rUTP + 0.023904 dATP + 0.024582 dGTP + 0.024582 dCTP + 0.023904 dTTP + 0.28352 mit_FS + 0.0069264 UDPGlc + 0.010368 CDPEth + 0.010368 OH_myr_ac + 0.010368 C14_0_FS + 0.010368 CMP_KDO + 0.010368 NDPHep + 0.0069264 TDPGlcs + 0.01656 UDP_NAG + 0.01656 UDP_NAM + 0.01656 di_am_pim + 0.0924 ADPGlc = Biomass .

O2_up : 1 O2_ext = 1 O2 .

N_up : 1 N_ext = 1 N .

CO2_ex : 1 CO2 = 1 CO2_ext .

S_up : 4 ATP + 4 NADPH = 1 S .

Glc_PTS : 1 Gluc + 1 PEP = 1 G6P + 1 Pyr .

Glc_ATP : 1 Gluc + 1 ATP = 1 G6P .

DHAP::Glyc3P : 1 DHAP + 1 NADH = 1 Glyc3P .

Lac_ex : 1 Lac = 1 Lac_ext .

Lact_up : 1 Lact_ext = 1 Lact .

Lactase : 1 Lact = 2 Gluc .

FL_rxn : 1 G6P + 1 ATP + 1 NADPH + 1 Lact = 1 FL_ext .

Eth_ex : 1 Eth = 1 Eth_ext .

Ac_ex : 1 Ac = 1 Ac_ext .

Form_ex : 1 Form = 1 Form_ext .

G6P::F6P : 1 G6P = 1 F6P .

F16P::F6P : 1 F16P = 1 F6P .

F6P::F16P : 1 F6P + 1 ATP = 1 F16P .

F16P::T3P : 1 F16P = 1 DHAP + 1 G3P .

DHAP::G3P : 1 DHAP = 1 G3P .

G3P::DPG : 1 G3P = 1 DPG + 1 NADH .

DPG::3PG : 1 DPG = 1 3PG + 1 ATP .

3PG::2PG : 1 3PG = 1 2PG .

2PG::PEP : 1 2PG = 1 PEP .

PEP::PYR : 1 PEP = 1 Pyr + 1 ATP .

Pyr::PEP : 1 Pyr + 2 ATP = 1 PEP .

PYR::AcCoA : 1 Pyr = 1 AcCoA + 1 NADH + 1 CO2 .

AcCoA::Cit : 1 AcCoA + 1 OxA = 1 Cit .

Cit::ICit : 1 Cit = 1 ICit .

ICit::alKG : 1 ICit = 1 alKG + 1 NADPH + 1 CO2 .

alKG::SuccCoA : 1 alKG = 1 SuccCoA + 1 NADH + 1 CO2 .

SuccCoA::Succ : 1 SuccCoA = 1 Succ + 1 ATP .

Succ::Fum : 1 Succ = 1 Fum + 1 QH2 .

Fum::Succ : 1 Fum + 1 QH2 = 1 Succ .

Fum::Mal : 1 Fum = 1 Mal .

Mal::OxA : 1 Mal = 1 OxA + 1 NADH .

ICit::Glyox : 1 ICit = 1 Succ + 1 Glyox .

Glyox::Mal : 1 AcCoA + 1 Glyox = 1 Mal .

G6P::PGlac : 1 G6P = 1 PGlac + 1 NADPH .

AcCoA::Adh : 1 AcCoA + 1 NADH = 1 Adh .

Adh::Eth : 1 NADH + 1 Adh = 1 Eth .

PGlac::PGluc : 1 PGlac = 1 PGluc .

PGluc::Rl5P : 1 PGluc = 1 Rl5P + 1 NADPH + 1 CO2 .

Rl5P::X5P : 1 Rl5P = 1 X5P .

Rl5P::R5P : 1 Rl5P = 1 R5P .

Transket1 : 1 R5P + 1 X5P = 1 G3P + 1 S7P .

Transaldo : 1 G3P + 1 S7P = 1 F6P + 1 E4P .

Transket2 : 1 E4P + 1 X5P = 1 F6P + 1 G3P .

OxA::PEP : 1 OxA + 1 ATP = 1 PEP + 1 CO2 .

PEP::OxA : 1 PEP + 1 CO2 = 1 OxA .

AcCoA::AcP : 1 AcCoA = 1 AcP .

AcP::Ac : 1 AcP = 1 ATP + 1 Ac .

Pyr::Form : 1 Pyr = 1 AcCoA + 1 Form .

Pyr::Lac : 1 Pyr + 1 NADH = 1 Lac .

NADHDehydro : 1 NADH = 1 QH2 + 2 Hp .

Oxidase : 1 QH2 + 0.5 O2 = 2 Hp .

TransHydro : 1 NADH + 1 Hp = 1 NADPH .

ATPSynth : 3 Hp = 1 ATP .

ATPdrain : 1 ATP = 1 ATP_ext .

Chor_Synth : 2 PEP + 1 E4P + 1 ATP + 1 NADPH = 1 Chor .

PRPP_Synth : 1 R5P + 2 ATP = 1 PRPP .

MTHF_Synth : 1 ATP + 1 NADPH = 1 MTHF .

Ala_Synth : 1 Pyr + 1 Glu = 1 alKG + 1 Ala .

Val_Synth : 2 Pyr + 1 NADPH + 1 Glu = 1 alKG + 1 CO2 + 1 Val .

Leu_Synth : 2 Pyr + 1 AcCoA + 1 NADPH + 1 Glu = 1 alKG + 1 NADH + 2 CO2 + 1 Leu .

Asn_Synth_1 : 2 ATP + 1 N + 1 Asp = 1 Asn .

Asp_synth : 1 OxA + 1 Glu = 1 alKG + 1 Asp .

Asp::Fum : 1 Asp = 1 Fum + 1 N .

Asp::AspSAld : 1 ATP + 1 NADPH + 1 Asp = 1 AspSAld .

AspSAld::HSer : 1 NADPH + 1 AspSAld = 1 HSer .

Lys_Synth : 1 di_am_pim = 1 CO2 + 1 Lys .

Met_Synth : 1 SuccCoA + 1 MTHF + 1 HSer + 1 Cys = 1 Pyr + 1 Succ + 1 N + 1 Met .

Thr_Synth : 1 ATP + 1 HSer = 1 Thr .

Ile_Synth : 1 Pyr + 1 NADPH + 1 Glu + 1 Thr = 1 alKG + 1 CO2 + 1 N + 1 Ile .

His_Synth : 1 ATP + 1 PRPP + 1 Gln = 1 alKG + 2 NADH + 1 His .

Glu_synth : 1 alKG + 1 NADPH + 1 N = 1 Glu .

Gln_Synth : 1 ATP + 1 N + 1 Glu = 1 Gln .

Pro_Synth : 1 ATP + 2 NADPH + 1 Glu = 1 Pro .

Arg_Synth : 1 AcCoA + 4 ATP + 1 NADPH + 1 CO2 + 1 N + 1 Asp + 2 Glu = 1 alKG + 1 Fum + 1 Ac + 1 Arg

Trp_Synth : 1 Chor + 1 PRPP + 1 Gln + 1 Ser = 1 G3P + 1 Pyr + 1 CO2 + 1 Glu + 1 Trp .

Tyr_Synth : 1 Chor + 1 Glu = 1 alKG + 1 NADH + 1 CO2 + 1 Tyr .

Phe_Synth : 1 Chor + 1 Glu = 1 alKG + 1 CO2 + 1 Phe .

Ser_Synth : 1 3PG + 1 Glu = 1 alKG + 1 NADH + 1 Ser .

Gly_Synth : 1 Ser = 1 MTHF + 1 Gly .

Cys_Synth : 1 AcCoA + 1 S + 1 Ser = 1 Ac + 1 Cys .

rATP_Synth : 5 ATP + 1 CO2 + 1 PRPP + 2 MTHF + 2 Asp + 1 Gly + 2 Gln = 2 Fum + 1 NADPH + 2 Glu + 1 rATP .

rGTP_Synth : 6 ATP + 1 CO2 + 1 PRPP + 2 MTHF + 1 Asp + 1 Gly + 3 Gln = 2 Fum + 1 NADH + 1 NADPH + 3 Glu + 1 rGTP .

rCTP_Synth : 1 ATP + 1 Gln + 1 rUTP = 1 Glu + 1 rCTP .

rUTP_Synth : 4 ATP + 1 N + 1 PRPP + 1 Asp = 1 NADH + 1 rUTP .

dATP_Synth : 1 NADPH + 1 rATP = 1 dATP .

dGTP_Synth : 1 NADPH + 1 rGTP = 1 dGTP .

dCTP_Synth : 1 NADPH + 1 rCTP = 1 dCTP .

dTTP_Synth : 2 NADPH + 1 MTHF + 1 rUTP = 1 dTTP .

mit_FS_Synth : 8.24 AcCoA + 7.24 ATP + 13.91 NADPH = 1 mit_FS .

UDPGlc_Synth : 1 G6P + 1 ATP = 1 UDPGlc .

CDPEth_Synth : 1 3PG + 3 ATP + 1 NADPH + 1 N = 1 NADH + 1 CDPEth .

OH_myr_ac_Synth : 7 AcCoA + 6 ATP + 11 NADPH = 1 OH_myr_ac .

C14_0_FS_Synth : 7 AcCoA + 6 ATP + 12 NADPH = 1 C14_0_FS .

CMP_KDO_Synth : 1 PEP + 1 R5P + 2 ATP = 1 CMP_KDO .

NDPHep_Synth : 1.5 G6P + 1 ATP = 4 NADPH + 1 NDPHep .

TDPGlcs_Synth : 1 F6P + 2 ATP + 1 N = 1 TDPGlcs .

UDP_NAG_Synth : 1 F6P + 1 AcCoA + 1 ATP + 1 Gln = 1 Glu + 1 UDP_NAG .

UDP_NAM_Synth : 1 PEP + 1 NADPH + 1 UDP_NAG = 1 UDP_NAM .

di_am_pim_Synth : 1 Pyr + 1 SuccCoA + 1 NADPH + 1 AspSAld + 1 Glu = 1 alKG + 1 Succ + 1 di_am_pim .

ADPGlc_Synth : 1 G6P + 1 ATP = 1 ADPGlc .

Mal::Pyr : 1 Mal = 1 Pyr + 1 NADH + 1 CO2 .

Pyr::Ac : 1 Pyr = 1 QH2 + 1 CO2 + 1 Ac .

Ac::AcCoA : 2 ATP + 1 Ac = 1 AcCoA .
